# The monoclonal antibody Ca37, developed against *Candida albicans* alcohol dehydrogenase, inhibits the yeast *in vitro* and *in vivo*

**DOI:** 10.1038/s41598-020-65859-4

**Published:** 2020-06-08

**Authors:** Aitziber Antoran, Leire Aparicio-Fernandez, Aize Pellon, Idoia Buldain, Leire Martin-Souto, Aitor Rementeria, Mahmoud A. Ghannoum, Beth Burgwyn Fuchs, Eleftherios Mylonakis, Fernando L. Hernando, Andoni Ramirez-Garcia

**Affiliations:** 1 Fungal and Bacterial Biomics Research Group. Department of Immunology, Microbiology and Parasitology. Faculty of Science and Technology, University of the Basque Country (UPV/EHU), Leioa, Spain; 2 Department of Dermatology and Center for Medical Mycology, Case Western Reserve University, and University Hospitals Cleveland Medical Center, Cleveland, Ohio USA; 3 Division of Infectious Diseases, Rhode Island Hospital, Warren Alpert Medical School of Brown University, Providence, Rhode Island, USA; 4 Present Address: Aize Pellon, Centre for Host-Microbiome Interactions, Mucosal and Salivary Biology Division, King’s College London Dental Institute, London, United Kingdom

**Keywords:** Biological techniques, Microbiology

## Abstract

*Candida albicans* is a commensal yeast able to cause life threatening invasive infections particularly in immunocompromised patients. Despite the availability of antifungal treatments, mortality rates are still unacceptably high and drug resistance is increasing. We, therefore, generated the Ca37 monoclonal antibody against the *C. albicans* alcohol dehydrogenase (Adh) 1. Our data showed that Ca37 was able to detect *C. albicans* cells, and it bound to Adh1 in yeast and Adh2 in hyphae among the cell wall-associated proteins. Moreover, Ca37 was able to inhibit candidal growth following 18 h incubation time and reduced the minimal inhibitory concentration of amphotericin B or fluconazole when used in combination with those antifungals. In addition, the antibody prolonged the survival of *C. albicans* infected-*Galleria mellonella* larvae, when *C. albicans* was exposed to antibody prior to inoculating *G. mellonella* or by direct application as a therapeutic agent on infected larvae. In conclusion, the Ca37 monoclonal antibody proved to be effective against *C. albicans*, both *in vitro* and *in vivo*, and to act together with antifungal drugs, suggesting Adh proteins could be interesting therapeutic targets against this pathogen.

## Introduction

*Candida albicans* is a commensal microorganism typically present in the mucosal and gastrointestinal tracts of humans. However, in some cases, it can become pathogenic, causing diseases that range from superficial mucosal infections to invasive candidiasis, which comprises bloodstream infection (candidemia) and/or deep-seated candidiasis^1^. *Candida* spp. are among the most isolated pathogens in hospital settings and in fact, *C. albicans* is the fungus most associated with nosocomial infections, with a mortality rate that can reach up to 50%^2^. Due to the increasing number of immunocompromised patients and the aging of the population, it is tempting to speculate that in the following years infections due to this fungus may increase.

Currently, only four antifungal classes are available against invasive candidiasis, and drug resistance has been described to all of them^3^. In fact, since the appearance of the echinocandins more than 10 years ago, no new class of antifungal drugs has been introduced. There is, therefore, an urgent need for new treatments to combat fungal infections^4^, and inhibitory antibodies are a promising avenue. Monoclonal antibodies (mAb) are being studied as a possible therapy for candidiasis and other fungal infections, either alone or in combination with antifungals^5–8^.

Our research group focused on the interaction between the host and *C. albicans*, and found several proteins related to yeast pathogenicity^9–11^. One of these, alcohol dehydrogenase (Adh), despite its metabolic role in the cytoplasm, has been identified as an allergen and antigen^12^ that is also located on the cell wall^13–15^, where it may play a role as an adhesin, binding the host’s fibronectin^16^ and serum plasminogen^17^. *C. albicans* Adh1 protein has also been associated with the promotion of cancer because it catalyzes the production of carcinogenic acetaldehyde^18–21^.

Therefore, considering the importance of the Adh1 of *C. albicans* and the fact that the homology between the human and fungal Adh1 is low (32% of identity, as compared by BLAST (https://blast.ncbi.nlm.nih.gov/Blast.cgi), a tool for gene and protein sequence alignment comparison), the aim of this work was to produce and study the effect of an anti-Adh1 (Ca37) mAb on the growth and viability of *C. albicans*, and to test its potential use in therapy.

## Results

### Characterization of the specificity of the Ca37 monoclonal antibody

**Figure 1:**
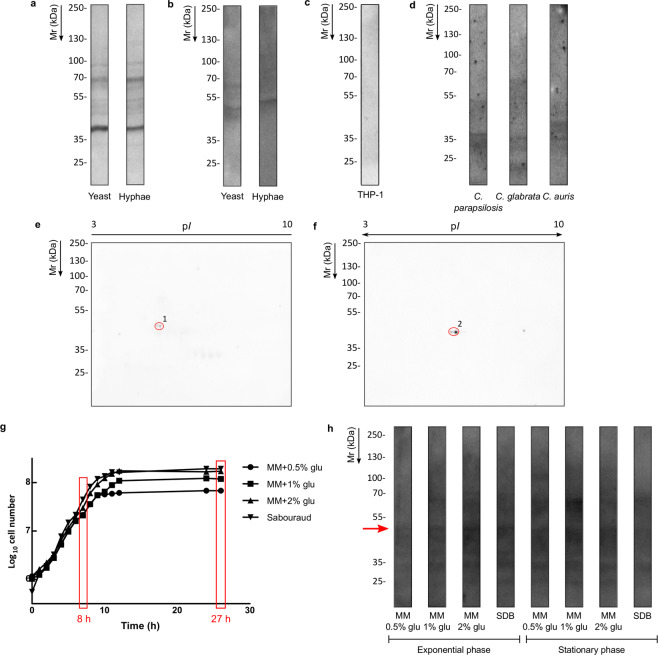
Western blotting of *Candida albicans* proteins recognized by Ca37 monoclonal antibody. SDS-PAGE western blotting of (**a**) cytosolic and (**b**) cell wall-associated proteins fractions of the fungus and (**c**) of THP-1 human monocyte cells. (**d**) Recognition of the Ca37 over the yeast cell wall-associated protein fraction of other *Candida* species. 2-DE western blotting of *C. albicans* (**e**) yeast and (**f**) hyphal cell wall-associated proteins. The identified spots are marked in red. (**g**) Growth curves of *C. albicans* using four different media: minimum media (MM) with different glucose concentrations (0.5%, 1% and 2%) and Sabouraud Dextrose broth (SDB). The time of yeast cell collection is marked in red. (**h**) Expression of the cell wall-associated proteins recognized by Ca37 in exponential and stationary phase grown in the mentioned four media. The red arrow marks the experimental molecular weight of the Adh1 protein. Blots have been cropped in order to facilitate the comparison among them; full-length blots are presented in Supplementary Figs. 1 and 2.

*C. albicans* specificity of the antibody was examined on cytosolic and cell wall-associated protein (CWP) fractions using both yeast and hyphae morphologies. In addition, the cross-reactivity with human cells was studied using a soluble protein fraction of the human monocytes THP-1 cell line, and the ability of the mAb to recognize other *Candida* genus species was also tested. The western blot (WB) performed over the sodium dodecyl sulfate polyacrylamide gel electrophoresis (SDS-PAGE) membrane showed a very similar banding pattern between the cytosolic fractions of both fungal yeast and hyphal morphologies (Fig. 1a), whereas the pattern of CWP fraction was different between yeasts and hyphae (Fig. 1b). The Ca37 antibody did not bind to any THP-1 cytosolic protein (Fig. 1c), and regarding the ability to bind other *Candida* species, the mAb recognized bands from *Candida parapsilosis*, *Candida glabrata* and *Candida auris* CWP fraction (Fig. 1e), although the pattern was different in each species. Only *C. albicans* yeast and hyphal CWP fractions were used for two-dimensional electrophoresis (2-DE) and WB analysis, because of the importance of this fraction owing to its closer association with the surrounding medium. In this fraction, the most immunoreactive spots were identified as alcohol dehydrogenase (Fig. 1e,f). However, the most immunoreactive spot from the yeast extract was identified as Adh1, whereas the one in hyphae extract was determined to be Adh2 (Table 1).

**Table 1:** LS/MS-MS identification of the protein spots detected by Ca37 monoclonal antibody in the cell wall-associated protein fraction western blots.

Spot no.	Accesion no.	Protein name	Matching peptides	Coverage (%)	MASCOT score	Theor. p*I*/Mr	Exper. p*I*/Mr
1	P43067.1	Alcohol dehydrogenase 1	5	16	368	6.02/37	5.1/46
2	KGU32026.1	Alcohol dehydrogenase 2	2	6	128	6.25/37	6.31/40.5

On the other hand, to test the expression of the *C. albicans* Adh1 in different media and growth phases, CWP from yeasts collected in exponential (8 h) and stationary phases (27 h) using 4 different media (minimal medium (MM) supplement with 0.5%, 1% and 2% glucose and Sabouraud Dextrose Broth (SDB), Fig. 1g) were analysed (Fig. 1h). Adh was present in all the texted conditions. A differential expression of some bands was observed in different conditions, being, in general, more reactive in stationary phase, but no significant differences were found. The original WB of all the blots presented in Fig. 1 can be found in Supplementary Figs. 1 and 2.

**Figure 2:**
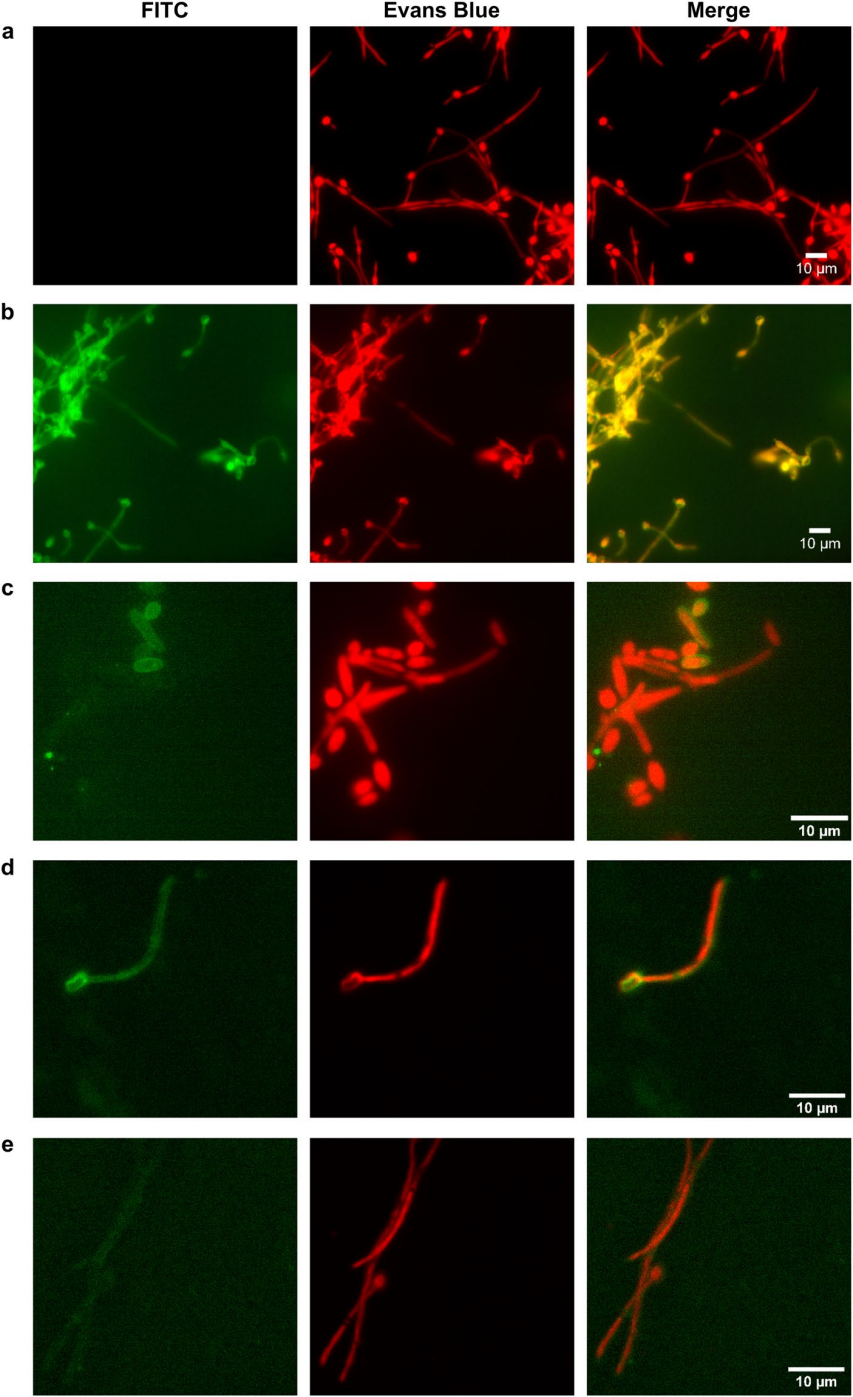
Visualization of the Ca37 monoclonal antibody-mediated recognition of *Candida albicans* yeast and hyphae through indirect immunofluorescence. *C. albicans* NCPF 3153 strain incubated (**a**) for 1.5 h with the Ca37 monoclonal antibody, (**b**) for 1.5 h with the Ca37 monoclonal antibody after treatment with 50 mM sodium metaperiodate and (**c**) for 18 h with the Ca37 monoclonal antibody. *C. albicans ADH1* knockout strain incubated (**d**) for 1.5 h with the Ca37 monoclonal antibody after treatment with 50 mM sodium metaperiodate and (**e**) for 18 h with the Ca37 monoclonal antibody.

In addition, an indirect immunofluorescence (IIF) assay was carried out to study whether the fungal proteins identified on the CWP fraction were accessible to antibody recognition on the cell surface (Fig. 2). Ca37 bound to *C. albicans* only when cells were pre-treated with sodium metaperiodate or when the incubation time was extended to 18 h, indicating that the mAb needs the elimination of the carbohydrates achieved by oxidation with metaperiodate or extended incubation times in order to penetrate the carbohydrate layer of the cell wall and detect the Adh proteins. In the second case (18 h incubation), the antibody was able to bind both yeast and hyphal cells, although not every cell was bound by the mAb, nor was the entire cell surface covered by the mAb (Fig. 2c), indicating low epitope availability. We also performed the IIF assay over an *ADH1* null mutant strain^22^, but in both cases (with metaperiodate treatment and extended time of incubation) a recognition of the Ca37 mAb was observed (Fig. 2d,e, respectively) in a similar way than in its wild type (data not shown) and the control strain.

### *In vitro* assessment of Ca37 antibody

**Figure 3:**
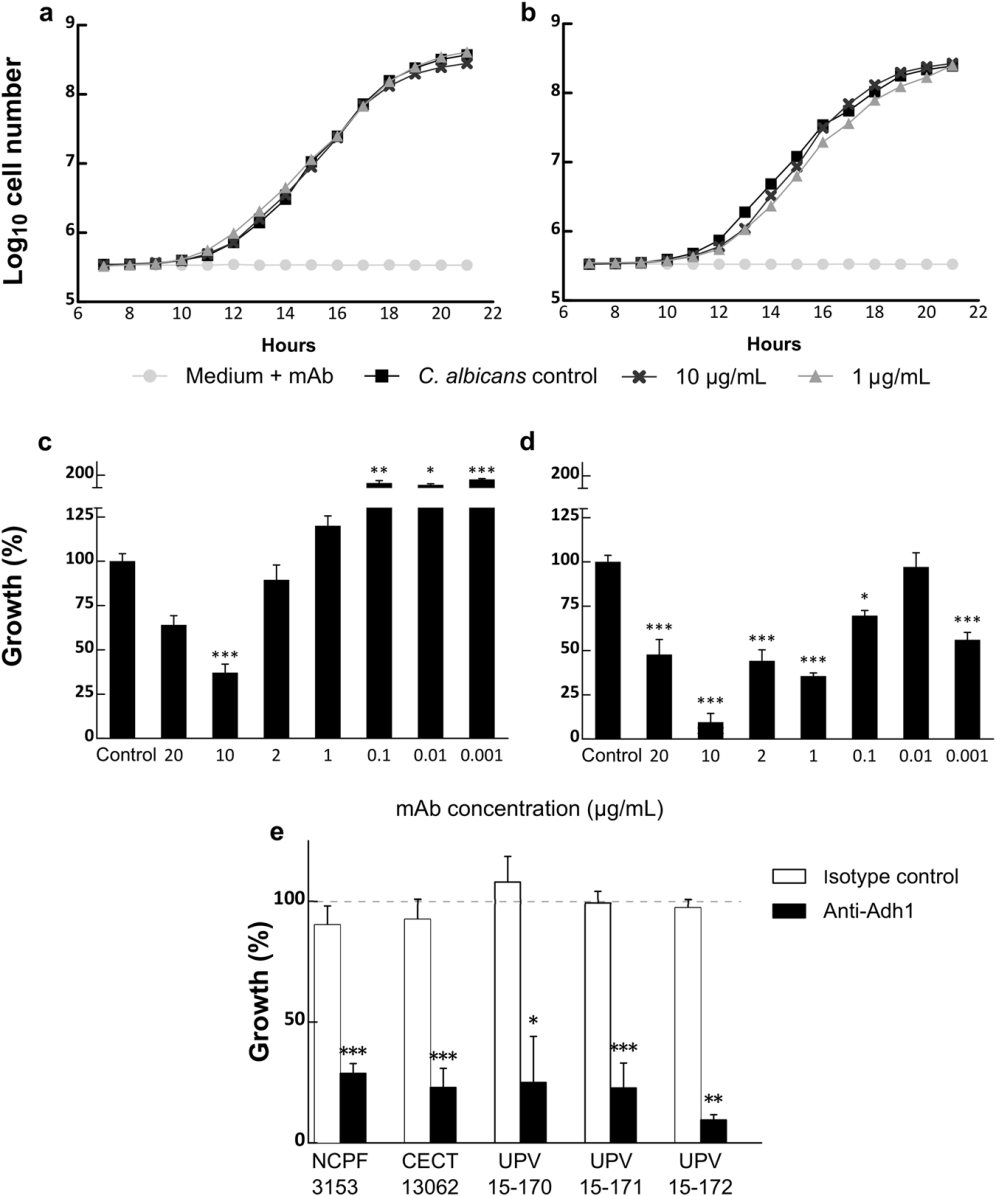
Effect of the Ca37 monoclonal antibody on *Candida albicans* growth. Growth curves of (**a**) NCPF 3153 strain and (**b**) CECT 13062 strain treated with Ca37 mAb, measured at 600 nm. The number of mAb concentrations studied have been reduced in order to allow a better visualization of the results. Growth inhibition of (**c**) NCPF 3153 strain (*P* values were 0.108 for 20 μg/mL, 0.001 for 10 μg/mL, 1 for 2 and 1 μg/mL, 0.002 for 0.1 μg/mL, 0.016 for μg/mL and 0 for 0.001 μg/mL) and (**d**) CECT 13062 strain (*P* values were <0.0001 for 20, 10, 2, and 1 μg/mL, 0.037 for 0.1 μg/mL, 1 for 0.01 μg/mL and 0.001 for 0.001 μg/mL) in presence of a wide range of Ca37 mAb concentrations, and (**e**) growth of five *C. albicans* strains were inhibited by 10 μg/mL Ca37 mAb. *P* values were <0.0001 for NCPF 3153 and CECT, 0.02 for UPV 15–170, 0.001 for UPV 15–172 and 0.003 for UPV 15–172. A non-specific IgG1 isotype control was included. Results are presented as percentage of growth in comparison with the control (100%). N = 3 in all cases. All data were normally distributed, therefore one way ANOVA was used with Bonferroni’s (for homogenous variances) or Tamhane’s T2 (for non-homogenous) corrections for multiple comparisons. Alpha value was set to 0.05 at least. Statistically significant differences in comparison to the non-treated control are marked as *P ≤ 0.05, **P ≤ 0.01, ***P ≤ 0.001.

After confirming antibody recognition, the effect on *C. albicans* NCPF 3153 and CECT 13062 strains growth was tested. Data showed that exposure to Ca37 had no significant effect on fungal growth (Fig. 3a,b). It is possible that active yeast division could hamper the action of the antibody during growth; therefore, inhibition was also studied by incubating *C. albicans* with the mAb in Dubelcco’s phosphate buffer saline (DPBS) prior to counting the colony-forming units (CFUs) grown in agar plates. In this case, a significant reduction in *C. albicans* CFU numbers was observed, 10 µg/mL being the most effective mAb concentration, which achieved a maximum of 90% inhibition (*P* = 0.001 in the case of NCPF 3153 strain and *P* < 0.0001 in the case of CECT 13062) (Fig. 3c,d). Therefore, this concentration was used with the rest of the strains, achieving an inhibition of between 70 to 90%, while the immunoglobulin (Ig) G1 isotype control exhibited no effect (Fig. 3e). The effect of Ca37 on yeast germination was also analysed. Although only three of the five strains showed a significant inhibition, the germination of all strain was inhibited between 6 and 20% (Table 2).

**Table 2:** Germination inhibition (in percentage) of five different *Candida albicans* strains in the presence of 20 and 10 μg/mL of Ca37 monoclonal antibody.

	20 µg/mL	10 μg/mL
NCPF 3153	6.11 (±1.35)	8.35 (±1.34)*
CECT 13062	9.31 (±2.73)	10.18 (±3.34)
UPV 15–170	17.38 (±0.35)*	9.06 (±4.41)
UPV 15–171	16.20 (±1.48)**	9.92 (±2.56)*
UPV 15–172	19.11 (±3.61)	18.58 (±7.12)

Statistically significant differences in comparison to control (no mAb added), *P* value for NCPF 3153 (10 μg/mL) was 0.02, for UPV 15–170 (20 μg/mL) 0.013 and for UPV 15–171 0.002 in the case of 20 μg/mL and 0.02 in the case of 10 μg/mL. N = 3 in all cases. All data were normally distributed, therefore one way ANOVA was used with Bonferroni’s (for homogenous variances) or Tamhane’s T2 (for non-homogenous) corrections for multiple comparisons. Alpha value was set to 0.05 at least.*P ≤ 0.05, **P ≤ 0.01.

**Figure 4:**
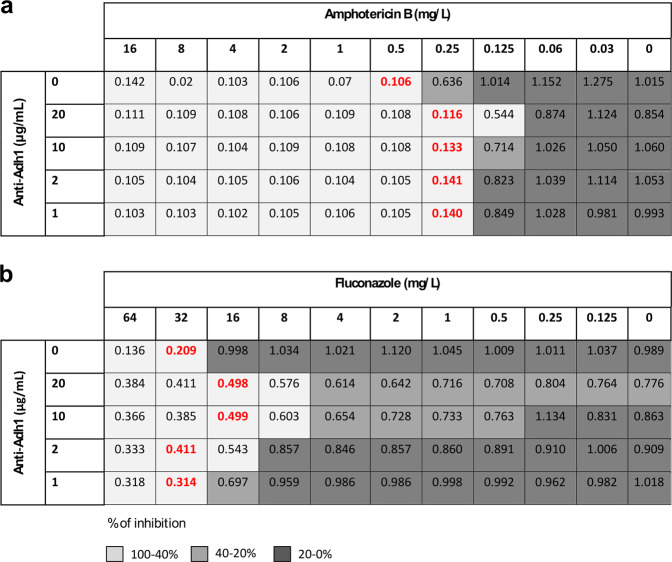
Additive effect of Ca37 monoclonal antibody with antifungals. (**a**) CECT 13062 strain and amphotericin B and (**b**) UPV 93–153 strain and fluconazole were used. MIC values are marked in red. The grey scale indicates the rate of inhibition, being higher in the case of light grey (100 to 40% of inhibition) and lower in the dark one (20 to 0% of inhibition).

Since Ca37 demonstrated inhibitory activity towards *C*. *albicans*, a possible synergy with current standard of care antifungal agents was investigated. The combined effects with antifungals that target different structures was explored: amphotericin B inhibits ergosterol synthesis and fluconazole targets cytochrome P450. Although the CECT 13062 strain was already susceptible to amphotericin B (The European Committee on Antimicrobial Susceptibility Testing-EUCAST breakpoints for antifungals, v. 9.0) the addition of the antibody reduced the minimal inhibitory concentration (MIC) value of amphotericin B to half, from 0.5 to 0.25 mg/L (Fig. 4a). In the case of fluconazole, the concentration of the antifungal needed to inhibit 50% of the growth of UPV 93–153 fluconazole-resistant strain was also reduced by half (from 32 to 16 mg/L) in the presence of 20 and 10 µg/mL of Ca37 (Fig. 4b). Fractional inhibitory concentration index (FICI) values were 0.55 for the combination of the anti-Adh1 with amphotericin B and 1 with fluconazole, both cases showing an additive effect.

### Effect of the Adh1 on fungal virulence, and effect of Ca37 antibody against the yeast *in vivo*

**Figure 5:**
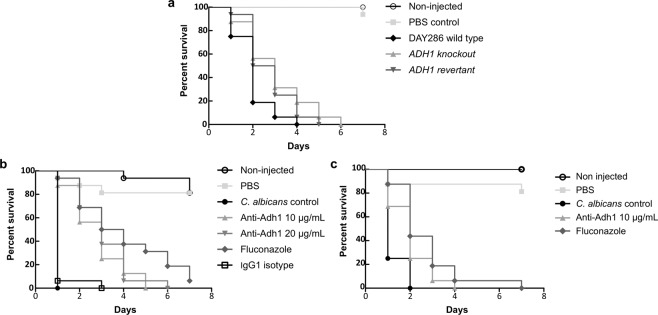
Effect of *ADH1* gene deletion and Ca37 monoclonal antibody on *Galleria mellonella* survival. (**a**) Larvae inoculated with DAY286, *ADH1* knockout and *ADH1* revertant *C. albicans* strains. *P* value was 0.0198 when comparing DAY286 with knockout and 0.0350 when comparing it with revertant strain. (**b**) *C. albicans* yeast were pre-treated with the mAb, fluconazole or a non-specific IgG1 isotype control. Yeast cells were pre-treated/incubated with 20 and 10 μg/mL of the mAb, 10 μg/mL of IgG1 isotype, fluconazole, or PBS alone (*C. albicans* control) for 18 h at 37 °C before been injected into larvae. Significant differences were found between *C. albicans* control and mAb pre-treated *C. albicans* groups (*P* < 0.001 in both cases). (**c**) Larvae treated with PBS (*C. albicans* control), 10 μg/mL Ca37 antibody or fluconazole after *C. albicans* infection (double injection). Significant differences were found between *C. albicans* control and antibody treatment (*P* = 0.0052) or fluconazole treatment (*P* < 0.001). Data from a single representative experiment (n = 16). Data were analysed using the Mantel-Cox test in GraphPad Prism software. Alpha value was set to 0.05. Non-injected and PBS injected larvae were used as viability control.

The role of *C. albicans* Adh1 in virulence and the ability of the Ca37 antibody to inhibit this pathogen were tested *in vivo* using *Galleria mellonella* as an invertebrate infection model (Fig. 5). To establish the infection profile of an *ADH1* mutant strain, larvae were initially infected with an *ADH1* knockout strain, which was found to be less virulent, with prolonged survival compared to the DAY286 parental strain (*P* = 0.0198; Fig. 5a). The larvae infected with the *ADH1* single allele revertant strain also showed an increased survival rate (*P* = 0.035) compared to the parental strain.

To study the inhibitory effect of the antibody, *C. albicans* (CECT 13062) cells were pre-treated with Ca37 before their inoculation. A fluconazole control and an IgG1 isotype control were also included (Fig. 5b). Data showed that the antibody was able to significantly reduce the mortality of *C. albicans* infected larvae (*P* = 0.0001), the mean survival time being 4–5 days longer than the *G. mellonella* group receiving non-treated yeast. The effect on the survival of the highest Ca37 concentration was only one half-day shorter than that of fluconazole. As expected, the IgG1 isotype control did not have any effect (*P* = 0.3173).

To test the therapeutic effect of Ca37, the mAb was injected immediately after infecting the larvae with *C. albicans*. As seen in Fig. 5c, *G. mellonella* treated with Ca37 or fluconazole survived a day longer than those larvae not treated with the mAb after *C. albicans* inoculation. The difference between non-treated larvae and larvae treated with Ca37 was statistically significant (*P* = 0.0052). Thus, showing that that the mAb was able to inhibit the *C*. *albicans* infection in the infection model, an encouraging finding.

## Discussion

Fungi present a particularly challenging pathogen to combat given that, like human cells, they are eukaryotic; and finding viable cellular targets that do not have adverse effects on the host is difficult. Proteins belonging to the cell wall of fungal pathogens have been studied as potential target for antifungal therapies, since this structure is not present in human cells. Alcohol dehydrogenase is a cytoplasmic enzyme, needed for glucose and ethanol processing, and plays an essential role in yeast metabolism; although, its role in the fungal cell wall is still unclear. It has been described previously as an antigen and adhesin^12,16^, but it may also play other roles yet to be elucidated. In fact, other metabolic proteins such as enolase have also been found on the cell wall and studied as therapeutic targets and even as a vaccine^23^.

Recently, monoclonal antibodies have been studied as alternative therapies in order to overcome the toxicity of current antifungal agents and the emergence of resistance to them. In fact, one anti-*Candida* mAb, named Mycograb, directed against heat shock protein 90, was developed, and showed a positive outcome in patients suffering from invasive candidiasis in combination with amphotericin B^24^. Unfortunately, due to concerns over its safety for use in humans, its application was discouraged in Europe^8^. This does not, however, mitigate the development of new mAbs against new or alternate fungal targets.

*C. albicans* Adh has recently been associated with a high immunogenic capacity and the conversion of monocytes into pro-inflammatory macrophages^25^. In addition, Adh inhibition may play a role in the reduction of fungal proliferation. Therefore, an anti-Adh1 monoclonal antibody, Ca37, was developed against *C. albicans* Adh1 protein and its effect on the viability of the yeast studied.

The mAb developed in this study specifically recognized Adh1 on yeast and Adh2 on hyphae CWP fraction. These two proteins share an 84% homology; therefore, the detection of these similar proteins is probably due to cross-reactivity. This result contrasts with those found by others, such as Pitarch *et al*.^13^, who found Adh1 in both yeast and hyphae CWP fraction, whereas Adh2 was detected in the cytosolic extract of hyphae^26^. In this study, we found that neither Adh1 nor any other human protein was detected by Ca37 antibody, as observed by western blot using THP-1 protein extract. This is not surprising, as the homology between human Adh1 protein and *C. albicans* Adh1 and Adh2 proteins is considerably low, 32% and 30%, respectively (BLAST alignment tool). This opens a possibility of using Ca37 mAb in humans once its efficacy is proven. Regarding the cross-reactivity with other *Candida* species, Ca37 mAb was able to recognize *C. parapsilosis, C. glabrata* and *C. auris*. As the homology between Adh protein of those species with *C. albicans* Adh proteins is high, between 73 to 85% (as analysed by BLAST) this fact was expected. The differences observed in the band pattern between the four species might be due to posttranscriptional modifications, because their theoretical molecular weight is similar (around 37 kDa for *C. albicans*, *C. glabrata* and *C. auris* and 43 kDa for *C. parapsilosis;* http://candidagenome.org), but this should be further studied. This result opens the door to study the use of the Ca37 mAb against other important *Candida* species, which could be especially critical in the case of *C. auris*, due to its high antifungal resistance profile^27^.

The expression pattern of Adh1 protein was also analysed in different media and growth phases, showing a slight, non-significant changes in expression depending on the medium and growth phase. These results agree with a previously reported study^28^, where a constant Adh1 expression was found by analyzing mRNA. Although not being great differences, our results show more reactivity in stationary phase of cells grown in some media, which may be related to the fact that Adh2 has been described to be expressed only in the stationary phase^28,29^. Although we used yeast CWP for the expression study, we cannot totally rule out the possibility that the mAb is also recognizing some of the Adh2 protein.

Surprisingly, the IIF did not show any recognition of the fungus over short incubation times unless there was prior removal of carbohydrates, suggesting that these molecules prevent mAb binding. However, after an incubation of 18 h the antibody was able to bind both fungal morphologies, although not uniformly. This may indicate that the epitope is partially obscured by carbohydrates, and considering the high immunogenicity recently described for this protein the hypothesis seems plausible^25^. On the other hand, the *ADH1* null mutant was recognized by the antibody in spite of its lack of Adh1 protein. The ability of the Ca37 to bind hyphae is not surprising, as we found that Adh2 is present in the CWP fraction of that morphology. However, the unexpected recognition of the yeasts would need further investigation to elucidate whether Adh2 might be overexpressed in order to compensate the absence of Adh1.

The activity of the mAb against *C. albicans* could not be observed on actively growing yeasts, but a small inhibition of 20% in germination was found. This contrasts with a recent study^30^ showing a decreased hyphal formation by an *ADH1* null mutant. However, in this article, the authors suggest that the inhibition of hyphal formation is due to a decreased intracellular ATP content and the suppression of a signalling pathway controlling various virulence factors, which would be related to the cytosolic role of the Adh1, whereas Ca37 antibody will presumably act only upon Adh1 protein present in the cell wall. On the contrary, when the yeast was incubated with the mAb in DPBS before inoculation onto Sabouraud Dextrose agar (SDA) plates, the inhibition percentages reached 70 to 90% compared to the control. Other authors have achieved similar rates of inhibition against *C. albicans* using monoclonal antibodies (63%, 74–92% and 55–100%), but the mAb concentration used was between 2.5 to 10 times higher than the concentration tested in this study^31–33^.

As the use of antifungal drugs at high doses can lead to hepatic and nephrotoxicity, synergistic ability between Ca37 and commonly used antifungals was also tested. It was found that the MIC values for amphotericin B and fluconazole were reduced to half in the strains used. Therefore, the antibody showed an additive effect, which might be of high relevance for clinicians, reducing high dose-associated adverse effects of antifungal drugs. It was shown that *ADH1* is upregulated in yeast treated with fluconazole^34^, which could explain the observed effect of the mAb. In the case of the fluconazole-resistant strain, this effect was not enough to reduce the *in vitro* MIC to a therapeutically successful concentration (2 mg/L or less^35^). However, the antibody might render some strains with intermediate susceptibility more sensitive to the action of this antifungal. Nevertheless, this concept needs to be tested and confirmed. Other studies have already shown that a combination of a mAb and an antifungal drug can enhance the survival of mice infected with *Candida*^5^.

In the *in vivo* assessment, it is worth noting that the fungal density used was much higher than the one used for the *in vitro* tests, but larvae possess hemocytes and antimicrobial peptides that help them overcome infections^36^, and to survive to high inoculums of *C. albicans* (10^4^ yeast/larva)^37^. In these assays, the importance of the Adh1 for fungal virulence was shown, as *G. mellonella* larvae infected with a *C. albicans ADH1* null mutant strain exhibited prolonged survival in comparison to the larvae infected with the parental strain. The group treated with the single allele revertant strain also lived longer than the group infected with the parental strain but it was slightly more pathogenic than the null mutant strain. In this strain, only one allele of the *ADH1* gene was reintroduced into the knockout mutant^22^, which may account for the failure to reach parental strain pathogenicity and fall as an intermediate. Song *et al*.^30^ also found that Adh1 is important for *C. albicans* pathogenicity, as they showed that survival of larvae, but also mice infected with an *ADH1* null mutant, was increase in comparison to the infected with the wild type strain.

Then, the ability of Ca37 to increase larvae survival was explored, both as pre-treatment of the yeasts before infection and as treatment for larvae. In both cases, an increase of *G. mellonella* survival was observed, by 5 days in larvae injected with pre*-*treated yeasts, and one day in mAb treated larvae after *C. albicans* inoculation, compared to larvae injected with the control fungus. This enhanced survival rate was similar to that observed with fluconazole treatment in both cases. It may also be that a higher or additional dose of the mAb may provide additional protection as a therapeutic. In the future, it would be interesting to study the additive effect of Ca37 with fluconazole, to determine whether a lower dose of drug could be protective in combination with the mAb, or whether *G. mellonella* infected with a fluconazole resistant strain could be recovered in the presence of Ca37.

Despite the promising results showed in this study, several questions should be addressed in the future. It would be interesting to deepen into the role of the Adh2 in virulence by constructing an *ADH2* and a double *ADH1/ADH2* null mutant, to elucidate whether the lack of Adh1 is compensated by Adh2, to study the effect of the Ca37 mAb in opsonization and phagocytosis, and to confirm the protective effect of the Ca37 mAb in a mice model.

In conclusion, the results presented here show that the Ca37 mAb, developed against Adh1 protein, is able to inhibit the growth of *C. albicans in vitro*, and to improve the survival of *G. mellonella in vivo*. Moreover, it acts in an additive way with amphotericin B and fluconazole, leading to a reduction of the MIC by half compared to the control. Therefore, although further research is needed to corroborate its efficacy in animal models and define its mechanism/s of inhibition, this study, together with studies of other authors, indicates that *C. albicans* Adh1 may represent an interesting therapeutic target and, as it does not bind to human Adh1 protein, that the Ca37 mAb is a candidate for further in-depth studies as an alternative and safe treatment.

## Methods

### Microorganisms and human cell line culture

Nine *Candida albicans* strains were used in this study: NCPF 3153, a reference strain; CECT 13062 strain, isolated from systemic candidiasis; UPV 93–153, a fluconazole resistant strain; UPV 15–170, UPV 15–171 and UPV 15–172 strains, isolated from oral candidiasis and kindly provided by Cristina Marcos-Arias from the Department of Immunology, Microbiology and Parasitology of the University of the Basque Country (UPV/EHU); and DAY286, *ADH1* knockout and revertant strains, obtained as described in Mukherjee *et al*.^22^. In addition to *C. albicans*, other three *Candida* species were used, *C. parapsilosis* ATCC 22019, *C. glabrata* ATCC 90030 (kindly provided by Dr. Dolores Moragues from the UPV/EHU) and *C. auris* CJ-197, isolated form an outbreak in La Fe Univeristy and Polytechnic Hospital (Valencia), which was a kind gift from Dr. Javier Pemán. The use of clinical strains from patients that had previously signed an anonymous informed consent form was approved by the Ethics Committee of the University of the Basque Country. All isolation methods were carried out in accordance with relevant guidelines and regulations.

For obtaining yeast cells, an overnight culture was inoculated with 10^5^ blastospores/mL in SDB and grown at 37 °C with agitation. Yeasts were always grown in this medium unless otherwise stated. For supporting the growth of the DAY286 and its derived *ADH1* knockout and revertant strains, histidine (100 µg/mL) was added to SDB. Hyphae were obtained by incubating yeast cells in RPMI 1640 supplemented with 10% fetal bovine serum (FBS) for 4 h at 37 °C with agitation.

THP-1 human monocyte cell line were obtained from the American Type Culture Collection and cultured in RPMI 1640 supplemented with 10% FBS and L-glutamine (2 mM), penicillin (10000 U/mL), streptomycin (10 mg/mL), amphotericin B (25 μg/mL) and β-mercaptoetanol (0.05 mM), in a 5% CO_2_ atmosphere at 37 °C. Subcultures were made when cell density reached 10^6^ viable cell/mL.

### Production of Adh1 recombinant protein and anti-Adh1 (Ca37) monoclonal antibody

The *C. albicans* CECT 13062 *ADH1* gene was cloned into pETBlue-2 vector (Novagen, Madison, WI, USA) and introduced into *Escherichia coli* Turner (DE3) pLac cells (Novagen). Protein expression was induced by addition of 1 mM IPTG and purification was performed by affinity chromatography (HisTrap FF crude kit, GE Healthcare, Chicago, IL, USA). Finally, the obtained Adh1 was treated with Polymyxin B for endotoxin removal.

The production of the anti-Adh1 Ca37 mAb was carried out in BALB/C mice by the hybridoma production technology at Abyntek Biopharma Company (Technology Park, Zamudio, Spain), using purified Adh1 for the immunization process. Briefly, splenocytes were fused with Sp2/0 myeloma cells and the most reactive clones were selected by enzyme-linked immunosorbent assay (ELISA). The antibodies were purified by protein G chromatography. For this study, an IgG1 isotype-producer hybridoma was selected.

In order to avoid antibody aggregates, the mAb was treated using a thermal-cycling method as in Sadavarte and Ghosh^38^ and filtered before use.

### Growth curves of yeast cells in different media

*C. albicans* CECT 13062 yeast cells where incubated at 10^6^ cell/mL in four different media; minimal medium supplemented with 0.5% glucose, 1% glucose and 2% glucose and in SDB. Minimal medium was composed of yeast nitrogen base (1.34%) and Biotin (0.02%). The yeasts were incubated at 37 °C and the 600 nm absorbance (Nanophotometer, Implen, Munich, Germany) was measured each hour. Absorbance data was translated into cell density. Yeast cells were harvested by centrifugation at 10,000 xg for 10 min at the middle of the exponential phase (8 h since the beginning of the incubation) and at the stationary phase (27 h since the beginning of the incubation). Yeast cell pellets were stored at −20 °C until further use.

### Protein extraction, electrophoresis (SDS-PAGE and 2-DE), western blotting and Liquid chromatography–mass spectrometry (LC-MS/MS) identification

Protein extraction was performed as described in Pitarch *et al*.^13^. Briefly, yeast or hyphal cells were mixed with glass beads and disrupted using a Millmix 20 BeadBeater cell disruptor (Tehtnica, Železniki, Slovenia) for 20 min at 30 Hz. Then, they were centrifuged in order to separate the cytosolic fraction (supernatant) from the cell wall (pellet). This pellet was treated with SDS extraction buffer for 10 min at 100 °C, then centrifuged and the supernatant was collected to obtain the CWP fraction. In the case of THP-1 cells, the followed procedure was similar, but with slight changes. Specifically, they were disrupted only for 2 minutes in the BeadBeater, centrifuged and only the soluble fraction was recovered for analysis.

For the SDS-PAGE, protein samples were loaded into 10% acrylamide gels and run at 70 mA, 100 W and 200 V for 45 min in a Miniprotean II (Bio-Rad, Hercules, CA, USA), using the Page Ruler Plus as protein standard (Thermo Fisher Scientific, Waltham, MA, USA). For 2-DE, the protocol described by Pellon *et al*.^39^ was followed, but using 7 cm long Immobiline Drystrip gels (GE Healthcare) for the isoelectric focusing (IEF), under the following conditions: rehydration for 12 h, 500 V for 2000 Vhr, 1000 V for 3000 Vhr, 5000 V for 10 000 Vhr, and 5000 V for 40000 Vhr. The proteins were then separated by SDS-PAGE. For protein identification, 2-DE gels were stained with Coomassie Brilliant Blue G250^40^.

To carry out the WB, proteins were transferred to Amersham Hybond-P PVDF membranes (GE Healthcare) for 1 h at 400 mA. Then, WB was performed using 30 μg/mL of Ca37 mAb at 37 °C for 1.5 h as primary antibody and an anti-mouse IgG-HRP diluted 1:100,000 for 45 min as secondary. Membranes were developed using the ECL prime (GE Healthcare) in a G:BOX Chemi system (Syngene, Cambridge, United Kingdom). All the incubation steps were carried out at room temperature unless otherwise indicated. The analysis of 2-DE and WBs was carried out by image analysis using ImageMaster 2DPlatinum Software (GE Healthcare).

The selected protein spots were manually excised from Coomassie Brilliant Blue G250 stained gels and identified by LC-MS/MS in the proteomics service of the UPV/EHU, SGIker, as described in Pellon *et al*.^39^.

### Indirect immunofluorescence

Fungal cells were used for detection using Ca37 by IIFs. They were carried out on cells treated with sodium metaperiodate (50 mM) to remove cell wall carbohydrates, and on non-treated cells. Then, 100 µg/mL of Ca37 mAb at 37 °C for 1.5 h or at 4 °C for 18 h were added as primary antibody, and anti-mouse IgG-FITC (4 μg/mL) diluted in PBS containing 0.05% (w/v) Evans Blue and 0.05% (v/v) Tween 20 for 30 min at 37 °C as secondary. Cells were observed under a fluorescent Eclipse N*i* microscope (Nikon, Tokio, Japan), and images taken with a Zyla sCMOS camera (Andor, Belfast, UK).

### Inhibitory effect of Ca37 monoclonal antibody on fungal growth and germination

For testing the effect of the antibody on the growth of *C. albicans*, 100 μL of 1.5 ×10^3^ yeast/mL in SDB were added to each well of a 96-well plate. SDB alone was used as blank and *C. albicans* without mAb was included as a growth control. Ten microliters of the antibody were added in a range from 10 μg/mL to 1 pg/mL. The plates were incubated at 37 °C and the absorbance at 600 nm measured every 10 min for 24 h (Synergy HT, BioTek, Winooski, VT, USA). Absorbance data from three different experiments were used and translated into densities.

To test the ability of the mAb to inhibit non-actively growing yeast, the procedure described by Mangliani *et al*.^41^ was followed. Briefly, 1.5 × 10^3^ yeast/mL were pre-incubated in DPBS for 18 h at 37 °C at 120 rpm with the same range of antibody concentrations mentioned above. Then, 100 μL of each treatment were inoculated onto SDA plates, in triplicate, so that the CFUs could be counted.

To study the effect of the Ca37 on *C. albicans* germination, suspensions of yeasts at 1.5 × 10^3^ yeast/mL were incubated in RPMI 1640 supplemented with 10% FBS, alone or with 20 or 10 µg/mL of mAb for 4 h at 37 °C and 120 rpm. Then, cells were fixed with 10% formalin saline and the percentage of germinated yeast was estimated by counting with an inverted optical microscopy.

### Measure of Minimal Inhibitory Concentration (MIC)

The effect of Ca37 mAb in combination with amphotericin B and fluconazole was studied following the guidelines of EUCAST^42^ using *C. albicans* CECT 13062 and fluconazole-resistant UPV 93–153 strains, and 20, 10, 2, 1 and 0 µg/mL concentrations of antibody.

The plates were incubated for 24 or 48 h at 37 °C, and absorbance recorded at 530 nm. The FICI was calculated using the derived MICs according to Bardbari *et al*.^43^.

### Insect infection studies

In this study *G. mellonella* infection model was used. Sixth-instar larvae (Grubco Company, Fairfield, OH, USA or Bichosa and Mundo Gusano, Vitoria, Spain) were injected with 10^6^ cells of *C. albicans* in 10 µL PBS through the last right pro-leg. Sixteen larvae were used per group, and two negative control groups were included: non-injected and PBS injected group. Larvae were incubated at 37 °C and checked daily over 7 days^44^. At least two independent experiments were performed (n ≥ 32).

Initially, DAY286, *ADH1* knockout and an *ADH1* revertant strain were used, following the procedure described above, to study the effect of the absence of the *ADH1* gene on yeast virulence. To observed the effect of the mAb, yeasts were treated for 18 h at 37 °C with Ca37 (20 and 10 μg/mL), a non-specific IgG1 isotype control from murine myeloma (Sigma-Aldrich, 10 μg/mL), fluconazole (13.86 μg/mL) or DPBS before using the treated *C. albicans* to inoculate *G. mellonella*. Conversely, we also studied the therapeutic effect of Ca37 by infecting larvae with *C. albicans* in the last right pro-leg followed by provision of 10 μL of the mAb (12 mg/kg), fluconazole (14 mg/kg) or PBS in the last left pro-leg. Once all the larvae were infected, *C. albicans* (pre- and not pre-treated) cells were inoculated into SDA in order to obtain a CFU count and make sure that 10^6^ cells/larva had been provided to all the groups.

### Statistical analysis

All data are presented as mean ± standard error. The statistical analyses were carried out using IBM SPPS statistics 22 (Professional Statistic, Chicago, IL, USA). Normality of data was checked using the Shapiro Wilks test and homogeneity of the variance using the Levene test. Normal data were analyzed using one-way ANOVA test (one-tailed test), with Bonferroni’s or Tamhane’s T2 corrections for multiple comparisons. In the case of the *G. mellonella* survival curves, Mantel-Cox test in GraphPad Prism software was used. Alpha value was set at 0.05, and the exact *P* values for each statistical analysis are showed in the text or figure legends.

## Supplementary information

Supplementary information.

## References

[1] Clancy CJ, Nguyen MH (2013). Finding the “missing 50%” of invasive candidiasis: How nonculture diagnostics will improve understanding of disease spectrum and transform patient care. Clin Infect Dis..

[2] Pfaller MA, Diekema D,J (2007). Epidemiology of invasive candidiasis: A persistent public health problem. Clin Microbiol Rev..

[3] Sanglard D (2016). Emerging threats in antifungal-resistant fungal pathogens. Front Med..

[4] Nami S, Aghebati-Maleki A, Morovati H, Aghebati-Maleki L (2019). Current antifungal drugs and immunotherapeutic approaches as promising strategies to treatment of fungal diseases. Biomed Pharmacother..

[5] Han Y (2010). Efficacy of combination immunotherapy of IgM MAb B6.1 and amphotericin B against disseminated candidiasis. Int Immunopharmacol..

[6] Mishra NN, Ali S, Shukla PK (2015). A monoclonal antibody against 47.2 kDa cell surface antigen prevents adherence and affects biofilm formation of *Candida albicans*. World J Microbiol and Biotechnol..

[7] Elguezabal N, Maza JL, Moragues MD, Pontón J (2009). Monoclonal antibody-mediated inhibition of adhesion of *Candida albicans* and *Candida dubliniensis* to human epithelial cells. Eur J Oral Sci..

[8] Bugli F (2013). Human monoclonal antibody-based therapy in the treatment of invasive candidiasis. Clin Dev Immunol..

[9] Hernando FL (2007). Identification of protein and mannoprotein antigens of *Candida albicans* of relevance for the serodiagnosis of invasive candidiasis. Int Microbiol..

[10] Ramirez-Garcia A (2011). Molecular fractionation and characterization of a *Candida albicans* fraction that increases tumor cell adhesion to hepatic endothelium. Appl Microbiol Biotechnol..

[11] Ramirez-Garcia A (2013). *Candida albicans* increases tumor cell adhesion to endothelial cells *in vitro*: Intraspecific differences and importance of the mannose receptor. PLoS One..

[12] Swoboda RK (1993). Glycolytic enzymes of *Candida albicans* are nonubiquitous immunogens during candidiasis. Infect..

[13] Pitarch A, Sánchez M, Nombela C, Gil C (2002). Sequential fractionation and two-dimensional gel analysis unravels the complexity of the dimorphic fungus *Candida albicans* cell wall proteome. Mol Cell Proteomics..

[14] Ebanks RO, Chisholm K, McKinnon S, Whiteway M, Pinto DM (2006). Proteomic analysis of *Candida albicans* yeast and hyphal cell wall and associated proteins. Proteomics..

[15] Castillo L (2008). A study of the *Candida albicans* cell wall proteome. Proteomics..

[16] Chaffin. WL, López-Ribot JL, Casanova M, Gozalbo D, Martínez JP (1998). Cell wall and secreted proteins of *Candida albicans*: identification, function, and expression. Microbiol Mol Biol Rev..

[17] Crowe JD (2003). *Candida albicans* binds human plasminogen: Identification of eight plasminogen-binding proteins. Mol Microbiol..

[18] IARC. A review of human carcinogens: chemical agents and related occupations. IARC Monogr Eval Carcinog risks to humans.;Vol. 100F:225–48 (2012). PMC4781612 23189753

[19] Tillonen J, Homann N, Rautio M, Jousimies-Somer H, Salaspuro M (1999). Role of yeasts in the aalivary acetaldehyde production from ethanol among risk groups for ethanol-associated oral cavity cancer. Alcohol Clin Exp Res..

[20] Uittamo J, Siikala E, Kaihovaara P, Salaspuro M, Rautemaa R (2009). Chronic candidosis and oral cancer in APECED-patients: Production of carcinogenic acetaldehyde from glucose and ethanol by *Candida albicans*. Int J Cancer..

[21] Ramirez-Garcia A (2016). *Candida albicans* and cancer: Can this yeast induce cancer development or progression?. Crit Rev Microbiol..

[22] Mukherjee PK (2006). Alcohol dehydrogenase restricts the ability of the pathogen *Candida albicans* to form a biofilm on catheter surfaces through an ethanol-based mechanism. Infect Immun..

[23] Li Wq (2011). Immunisation with the glycolytic enzyme enolase confers effective protection against *Candida albicans* infection in mice. Vaccine..

[24] Pachl J (2006). A randomized, blinded, multicenter trial of lipid-associated amphotericin B alone versus in combination with an antibody-based inhibitor of heat shock protein 90 in patients with invasive candidiasis. Clin Infect Dis..

[25] Liu Y (2019). Alcohol dehydrogenase of *Candida albicans* triggers differentiation of THP-1 cells into macrophages. J Adv Res..

[26] Hernández R, Nombela C, Diez-Orejas R, Gil C (2004). Two-dimensional reference map of *Candida albicans* hyphal forms. Proteomics..

[27] Bidaud. AL, Chowdhary A, Dannaoui E (2018). *Candida auris*: An emerging drug resistant yeast – A mini-review. J Mycol Med..

[28] Bakri. MM, Rich AM, Cannon RD, Holmes AR (2015). *In vitro* expression of *Candida albicans* dehydrogenase genes involved in acetaldehyde metabolism. Mol Oral Microbiol..

[29] Kusch H (2008). A proteomic view of *Candida albicans* yeast cell metabolism in exponential and stationary growth phases. Int J Med Microbiol..

[30] Song Y (2019). *ADH1* promotes *Candida albicans* pathogenicity by stimulating oxidative phosphorylation. Int J Med Microbiol..

[31] Kavishwar A, Shukla PK (2006). Candidacidal activity of a monoclonal antibody that binds with glycosyl moieties of proteins of *Candida albicans*. Med Mycol..

[32] Moragues MD (2003). A monoclonal antibody directed against a *Candida albicans* cell wall mannoprotein exerts three anti-*C. albicans* activities. Infect Immun..

[33] Polonelli L (2008). Antibody complementarity-determining regions (CDRs) can display differential antimicrobial, antiviral and antitumor activities. PLoS One..

[34] Copping VMS (2005). Exposure of *Candida albicans* to antifungal agents affects expression of SAP2 and SAP9 secreted proteinase genes. J Antimicrob Chemother..

[35] Pfaller MA (2012). Antifungal drug resistance: Mechanisms, epidemiology, and consequences for treatment. Am J Med..

[36] Mowlds P, Kavanagh K (2008). Effect of pre-incubation temperature on susceptibility of *Galleria mellonella* larvae to infection by *Candida albicans*. Mycopathologia..

[37] Cotter G, Doyle S, Kavanagh K (2000). Development of an insect model for the *in vivo* pathogenicity testing of yeasts. FEMS Immunol Med Microbiol..

[38] Sadavarte RH, Ghosh R (2014). A thermal-cycling method for disaggregating monoclonal antibody oligomers. J Pharm Sci..

[39] Pellon A (2016). Immunoproteomics-based analysis of the immunocompetent serological response to *Lomentospora prolificans*. J Proteome Res..

[40] Dyballa N, Metzger S (2009). Fast and sensitive colloidal coomassie G-250 staining for proteins in polyacrylamide gels. J Vis Exp JoVE..

[41] Mangliani W (1997). Therapeutic potential of antiidiotypic single chain antibodies with yeast killer toxin activity. Nat Biotechnol..

[42] Arendrup MC, Cuenca-Estrella M, Lass-Flörl C, Hope WW (2012). & EUCAST-AFST. Method for the determination of broth dilution minimum inhibitory concentrations of antifungal agents for yeasts. Clin Microbiol Infect..

[43] Bardbari AM (2018). Highly synergistic activity of melittin with imipenem and colistin in biofilm inhibition against multidrug-resistant strong biofilm producer strains of *Acinetobacter baumannii*. Eur J Clin Microbiol Infect Dis..

[44] Fuchs BB, O’Brien E, El Khoury JB, Mylonakis E (2010). Methods for using *Galleria mellonella* as a model host to study fungal pathogenesis. Virulence..

