# Supplementary material for: The monoclonal antibody Ca37, developed against Candida albicans alcohol dehydrogenase, inhibits the yeast in vitro and in vivo
Source: Sci Rep. 2020 Jun 8;10:9206. doi: 10.1038/s41598-020-65859-4 (PMC7280234; doi:10.1038/s41598-020-65859-4)
Supplement: Supplementary file 1 — Supplementary information. [file 41598_2020_65859_MOESM1_ESM.pdf]

SUPPLEMENTARY INFORMATION

The monoclonal antibody Ca37, developed against *Candida albicans* alcohol dehydrogenase, inhibits the yeast *in vitro* and *in vivo*

Aitziber Antoran[a], Leire Aparicio-Fernandez[a], Aize Pellon[a#], Idoia Buldain[a], Leire Martin-Souto[a], Aitor Rementeria[a], Mahmoud A. Ghannoum[b], Beth Burgwyn Fuchs[c], Eleftherios Mylonakis[c], Fernando L. Hernando*[a] and Andoni Ramirez-Garcia[a]

[a]Fungal and Bacterial Biomics Research Group. Department of Immunology, Microbiology and Parasitology. Faculty of Science and Technology. University of the Basque Country (UPV/EHU), Spain

[b]Center for Medical Mycology, Department of Dermatology, Case Western Reserve University and University Hospitals Cleveland Medical Center, Cleveland, Ohio, USA

[c]Division of Infectious Diseases, Rhode Island Hospital, Alpert Medical School of Brown University, Providence Rhode Island, USA

*Address correspondence to Fernando L. Hernando, fl.hernando@ehu.eus

#Present address: Aize Pellon, Centre for Host-Microbiome Interactions, Mucosal and Salivary Biology Division, King's College London Dental Institute, London, United Kingdom.

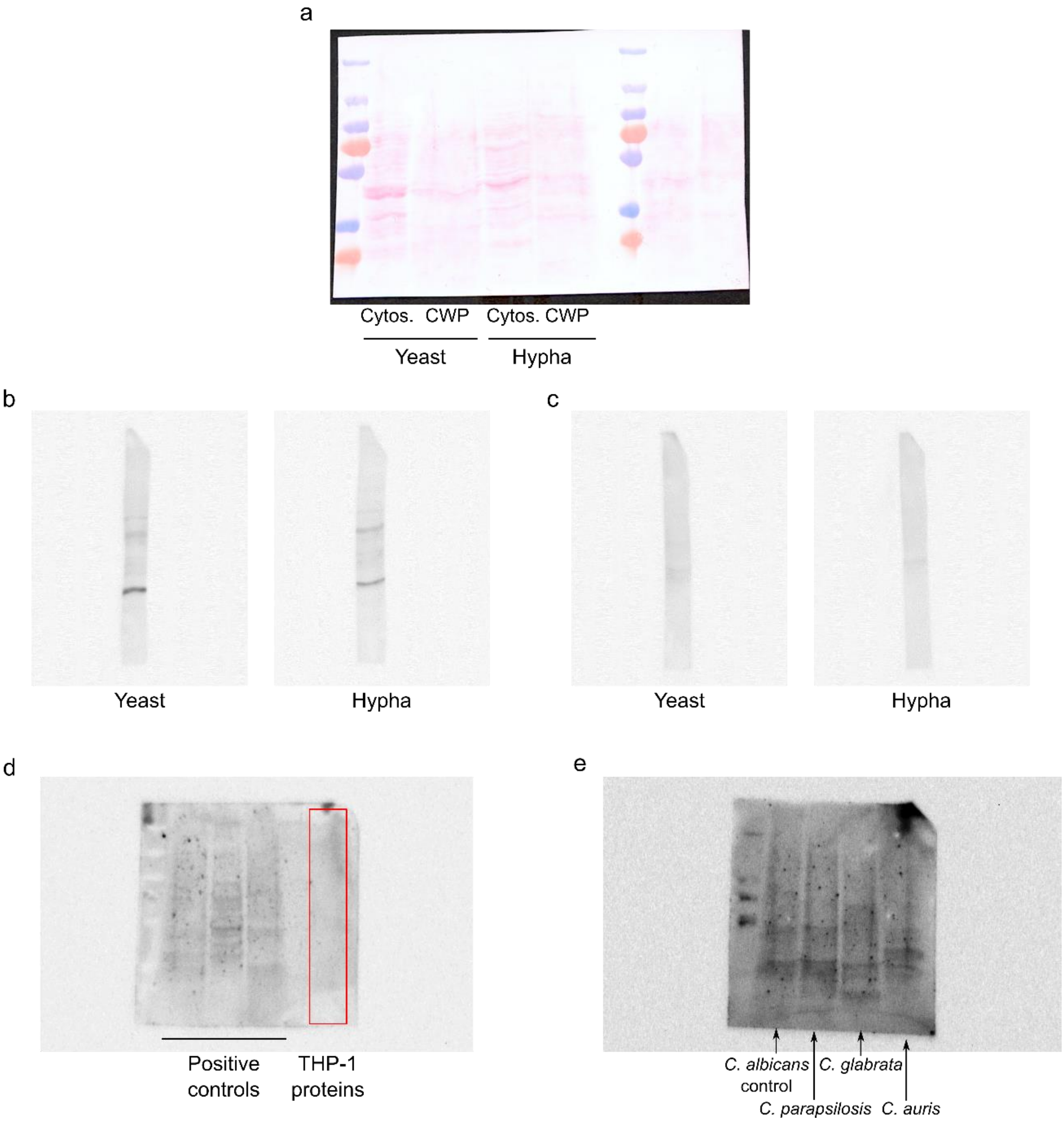

Supplementary figure 1. Original blots of the SDS-PAGE Western Blot from main text figure 1. a) The original membrane with fungal proteins, where molecular weight markers can be visualized. b) Fungal cytosolic proteins. c) Fugal cell wall-associated proteins. Each lane was cut before performing the WB, and therefore, incubated separately with the anti-Adh1 Ca37 monoclonal antibody, but all were revealed the same day and using the same exposure time. d) WB of the human mococyte THP-1 cell line soluble proteins. Three lanes containing fungal proteins were added as controls, the following lane was left empty, and in the last lane THP-1 proteins were loaded. e) Other *Candida* species cell wall proteins recognized by the Ca37 (lanes 3 to 5). In the second line a *C. albicans* cell wall protein control was added.

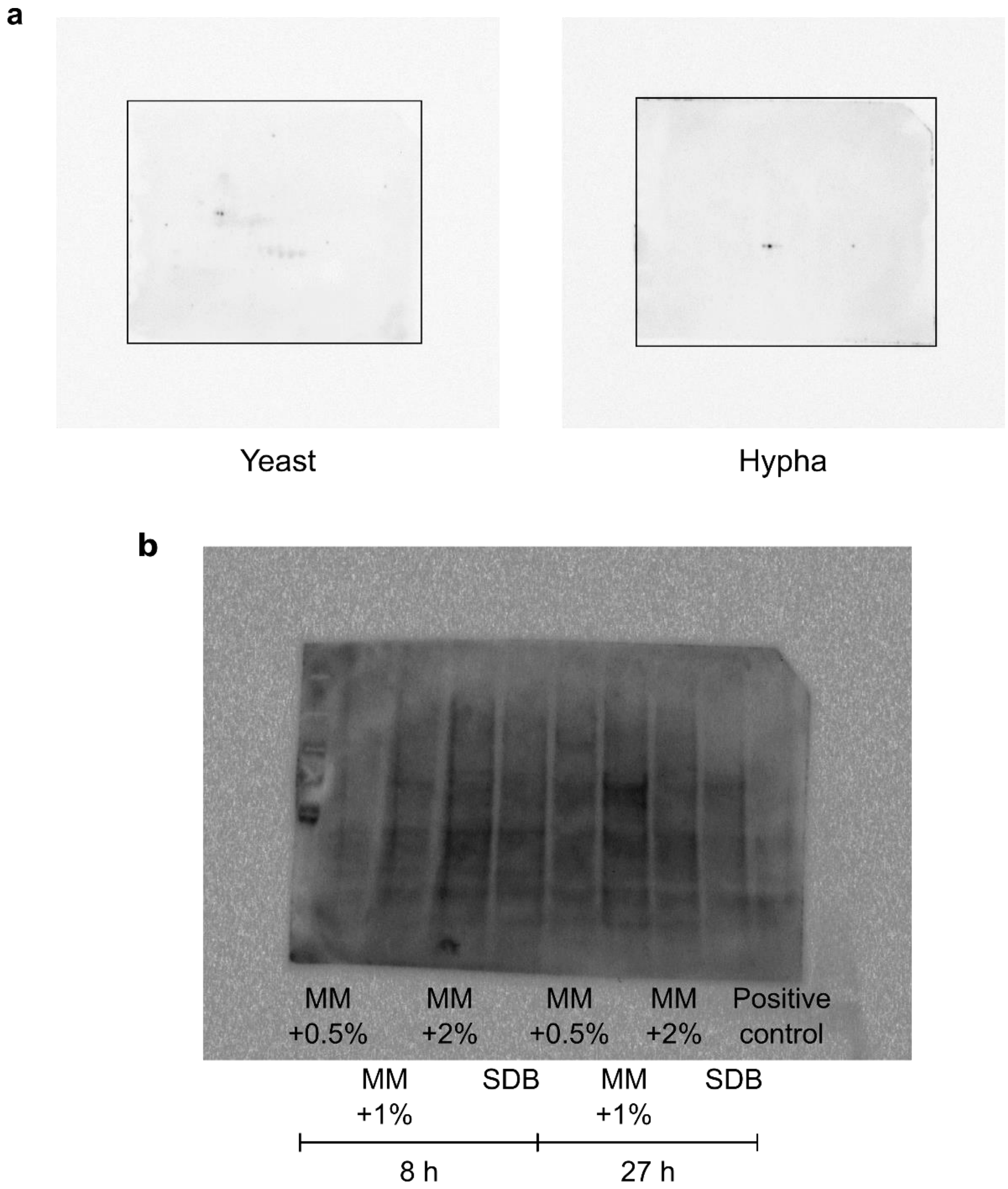

Supplementary figure 2. Original blot from the western blot performed over a two-dimensional membrane from figure 1 (e, and f subfigures) and the original western blot of the Adh expression analysis (subfigure 1h). a) Only cell wall-associated fraction was used to run the two-dimensional electrophoresis. Each membrane was incubated with the anti-Ah1 Ca37 antibody and revealed in a separate experiment. The revelation exposure time was 8 min for the yeast blot and 2 min for the hypha blot. b) Expression of the Adh cell wall-associated proteins recognized by the Ca37 antibody of *C. albicans* grown in different media (Minimal medium (MM) supplemented with 0.5%, 1% and 2% glucose and Sabouraud Dextrose broth, SDB) and extracted at different growth phases (8 h= exponential phase, 27 h = stationary phase). The last lane corresponds to a cell wall-associated protein control, used in the previous membranes.
